# Dr. George B. McCollum

**Published:** 1918-07

**Authors:** 


**Dr. George B. McCollum** of Buffalo died May 19, age 63. (Not listed in the N. Y. State Med. Directory).

